# Genome Silencing and Elimination: Insights from a “Selfish” B Chromosome

**DOI:** 10.3389/fgene.2017.00050

**Published:** 2017-04-25

**Authors:** John C. Aldrich, Patrick M. Ferree

**Affiliations:** W.M. Keck Science Department, Claremont McKenna College, Pitzer College and Scripps College, ClaremontCA, USA

**Keywords:** B chromosome, *Nasonia vitripennis*, histone modifications, heterochromatin, genome conflict

## Abstract

B chromosomes are non-essential components of numerous plant and animal genomes. Because many of these “extra” chromosomes enhance their own transmission in ways that are detrimental to the rest of the genome, they can be thought of as genome parasites. An extreme example is a paternally inherited B chromosome known as paternal sex ratio (PSR), which is found in natural populations of the jewel wasp *Nasonia vitripennis*. In order to ensure its own propagation, PSR severely biases the wasp sex ratio by converting diploid female-destined embryos into transmitting haploid males. This action occurs at the expense of the other paternally inherited chromosomes, which fail to resolve during the first round of division and are thus eliminated. Recent work has revealed that paternal genome elimination by PSR occurs through the disruption of a number of specific histone post-translational modifications, suggesting a central role for chromatin regulation in this phenomenon. In this review, we describe these recent advances in the light of older ones and in the context of what is currently understood about the molecular mechanisms of targeted genome silencing and elimination in other systems.

## Introduction

Many heritable elements present within eukaryotic genomes – for example, protein-coding genes—arise evolutionarily and persist because they confer some level of selective advantage to the organisms in which they reside. Such elements can be viewed as working together to enhance an organism’s fitness—in some cases being indispensable. Other elements, such as transposable elements, provide little or no fitness advantage and can behave in ways that are deleterious to the organism (Hurst and Werren, 2001; Werren, 2011).

An extreme example of a ‘genome parasite’ is the B chromosome PSR (paternal sex ratio) (Werren and Stouthamer, 2003). This diminutive (∼5 Mbp in size), extra chromosome is present at low levels in natural populations of the jewel wasp *Nasonia vitripennis* (Nur et al., 1988; Beukeboom and Werren, 2000). B chromosomes are broadly found in thousands of plant and animal genomes, and in most cases they carry few, if any, essential genes (Jones, 1991, 1995; Camacho et al., 2000). For this reason, B chromosomes are non-essential components of the genome and are prone to becoming lost in just a few cell divisions unless they can counter this tendency. PSR is unique in this regard: it is transmitted to new progeny solely via sperm and therefore must counter its own loss by drastically altering the wasp’s sex ratio to produce all male broods. This effect is initiated soon after fertilization when all of the paternal chromosomes, with the exception of PSR itself, are eliminated as the mitotic divisions of early embryogenesis begin (Werren et al., 1981, 1987; Swim et al., 2012). Elimination of half the genome in many organisms would be lethal. However, in *N. vitripennis* and all other hymenopteran insects—including all wasps, bees and ants—a half-genome equivalent is the normal signal for development into the male sex. Males normally develop from unfertilized haploid eggs while females of these insects develop from fertilized diploid eggs (Whiting, 1968). Thus, by eliminating the sperm’s hereditary material, PSR selfishly converts what should become diploid, female-destined embryos into males, the PSR-transmitting sex. This effect occurs with near perfect efficiency, resulting in all-male wasp broods that carry PSR (Beukeboom and Werren, 1993b). Thus, PSR is a genome parasite in the truest sense: it propagates at the expense of the wasp’s entire haploid paternal genome during every generation.

**FIGURE 1:**
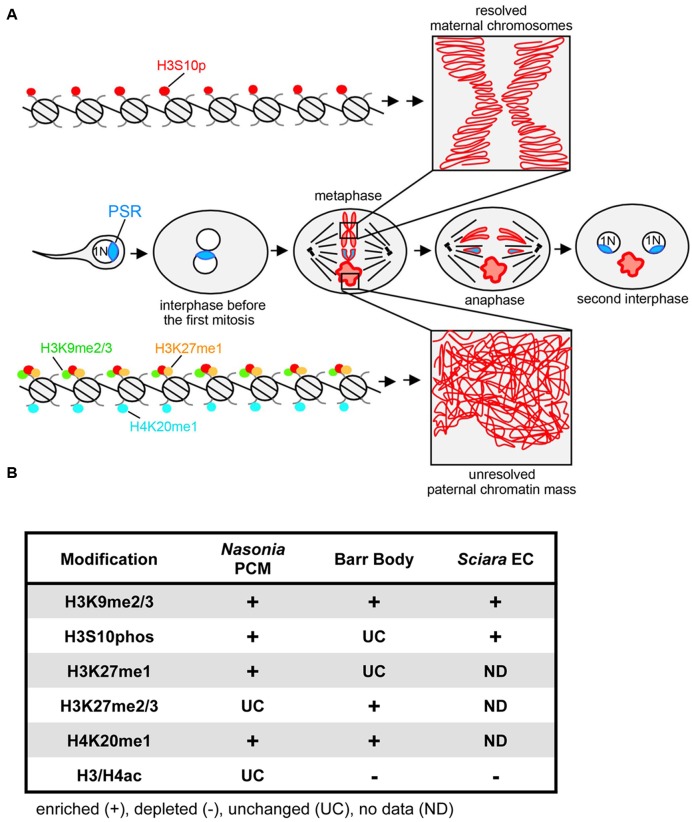
**PSR-induced chromatin modifications in *Nasonia vitripennis* and other genome silencing or elimination events. (A)** Schematic representation of histone post-translational modifications associated with PSR-induced genome elimination. In the center row, PSR is carried into the egg by the sperm. The two pronuclei come together and undergo one round of replication. As the two sets enter into the first mitosis, the maternal chromatin resolves normally into individual chromosomes while the paternal chromatin fails to resolve, becoming the paternal chromatin mass (PCM). The maternal chromosomes and the PSR chromosome, which is not blocked from resolving, segregate together, forming two haploid (1N) PSR+ nuclei. Top, a chromatin fiber from a region of euchromatin, which becomes phosphorylated at serine residue 10 of histone H3. This mark is associated with mitotic chromosome condensation (box in top right). Bottom, a chromatin fiber in euchromatin of the PCM. After fertilization, the paternal chromatin obtains abnormally high levels of di- and tri-methylated histone H3K9, mono-methylated histone H3K27, and mono-methylated histone H4K20. The H3S10p mark is also placed, but the combination of all four marks blocks normal chromosome resolution (box in bottom right). Chromatin marks that have been characterized on the *Nasonia* PCM are summarized in **(B)** along with what is known about these marks on the mammalian inactivated X chromosome (Barr body) (Hall and Lawrence, 2010) and the eliminated paternal chromosomes (EC) of *Sciara* (Goday and Ruiz, 2002; Greciano and Goday, 2006; Escribá et al., 2011). Symbols indicate that the specified modification is enriched (+), depleted (–), or unchanged (UC) on the eliminated or silenced chromosome(s) while “ND” indicates modifications for which there is no available data.

Induced paternal genome elimination is critical for propagation of PSR, and is arguably the most striking aspect of PSR’s biology. Early cytological studies showed that when PSR is present the paternal half of the genome becomes an abnormally compact mass (referred to as the paternal chromatin mass or PCM) that never transitions into individualized chromosomes during the first embryonic mitotic division following fertilization (Reed, 1993) (**Figure 1A**). This effect results in complete loss of the PCM at this earliest developmental stage. Superficially the PCM resembles heterochromatin–the compact, transcriptionally silent portion of the interphase genome – because it undertakes a similarly bright cytologically condensed appearance when visualized with certain DNA stains (Reed, 1993; Swim et al., 2012). These characteristics raise a number of important questions regarding paternal genome elimination by PSR. In particular, what is the molecular nature of the PCM and how are its properties different from normally functioning nuclear material? How does PSR cause PCM formation (i.e., genome elimination) at the molecular level while avoiding self-elimination? How does PSR target specific chromosomes and how is this targeting information conveyed? Here, we summarize findings from several recent studies that provide insights to these questions, emphasizing that PSR-induced genome elimination is a chromatin-based phenomenon and a prime example of conflict among elements within the same genome.

## Defining the Chromatin Basis of Genome Elimination

At its most basic level, chromatin consists of DNA packaged into a higher order state with the core histone proteins H2A, H2B, H3, and H4. These highly conserved proteins form an octamer—two of each histone—around which 146 bp of DNA is wrapped, together forming a structure known as the nucleosome, that is further arranged into higher order structures that package the genome. The histones’ N-terminal ‘tails’ are chemically modified at multiple amino acid residues in different ways including phosphorylation, methylation, and acetylation. Single modifications at individual residues or combinations of modifications at different residues can serve as interaction sites for chromatin-associated proteins including non-histone structural proteins and histone-modifying enzymes (Bannister and Kouzarides, 2011; van Steensel, 2011; Allis and Jenuwein, 2016). Association of these factors influences the higher order organization of chromatin, and therefore, characteristics such as its level of compactness and transcriptional activity. These properties are complex and dynamic, varying widely across different genome regions, stages of the cell cycle, and developmental periods (Schubeler et al., 2004; Ebert et al., 2006).

A handful of studies have sought to understand how the PCM forms by microscopically examining the chromatin state before, during, and immediately following the first embryonic mitosis (**Figure 1**). The first study of this kind addressed whether the PCM undergoes DNA replication (S-phase) and proper entry into the first mitotic division (Swim et al., 2012). By visualizing the active replication factor PCNA, it was found that the PCM successfully passes through the first S-phase. However, upon entry into mitosis the PCM becomes overloaded with condensins, proteins that mediate the transformation of decondensed chromatin into individualized chromosomes (Hirota et al., 2004; Hirano, 2005). This defect was found to correlate with an enrichment of phosphorylated histone H3 at Serine residue 10 (H3S10p) across the PCM. Additionally, both H3S10p and condensins fail to be removed properly from the PCM upon exit from the first mitosis. Previous work has suggested that proper loading of condensins to chromatin requires direct interaction of this complex with H3S10p (Wei et al., 1998, 1999; Giet and Glover, 2001). Together these observations imply that failure of the PCM to resolve into distinct chromosomes may be caused directly by abnormal overloading and retention of condensins, an effect that, in turn, may stem from abnormal retention of the H3S10p mark. Furthermore, these results raise the question of whether PSR alters H3S10p and condensin patterns directly or indirectly via some other earlier chromatin-related process.

Researchers recently addressed this question by examining additional histone marks that are placed earlier onto the paternal chromatin of PSR-carrying embryos (Aldrich et al., 2017). In many animals including insects, DNA of mature sperm is packaged with histone-like proteins known as protamines (Balhorn, 2007; Rathke et al., 2007). These proteins are removed from the sperm’s DNA immediately following fertilization and are simultaneously replaced with conventional histones. Work in Drosophila and other model organisms has shown that one of the first marks to appear on the repackaged paternal DNA is histone H4 acetylated at multiple Lysine residues (H4ac) (Loppin et al., 2005; Kanippayoor et al., 2013). In both wild type and PSR-carrying wasp embryos H4ac begins to appear on the ‘needle-shaped’ sperm nucleus as it moves toward the maternal nucleus, suggesting that PSR does not affect H4ac deposition. This result and the fact that the paternal chromatin progresses normally through the first S-phase together argue that PSR does not hinder protamine removal.

Unlike H4ac, which appears unaffected by PSR, three different marks, di- and tri-methylated histone H3 at Lysine 9 (H3K9me2/3), mono-methylated histone H3 at Lysine 27 (H3K27me1), and mono-methylated histone H4 at Lysine 20 (H4K20me1), were strikingly abnormal in this context (Aldrich et al., 2017). For example, in wild type embryos H3K9me2/3, a standard mark of constitutive (i.e., ‘permanent’) heterochromatin (Nakayam et al., 2001; Lehnertz et al., 2003), becomes visible in small regions of the wasp’s paternal nucleus soon after its juxtaposition with the maternal nucleus, and increases in intensity at these regions during mitosis. In contrast, when PSR is present H3K9me2/3 appears at the proper time but abnormally spreads across the entire paternal nucleus and persists as long as the PCM is visible. H3K27me1 and H4K20me1 have similar, abnormal patterns but, unlike H3K9me2/3, have less clear associations with heterochromatin. In mammals, both are associated with actively transcribed promoters (Li et al., 2011; Steiner et al., 2011; Ferrari et al., 2014) while H4K20me1 can be found in some silent regions including the inactive X (or Barr body) (Sims et al., 2006).

Rather than being a result of abnormal heterochro matinization, the hyper-condensed nature of the PCM may instead reflect a more complex, disorganized state of chromatin condensation that forms during attempted chromosome resolution. In other words, the alteration of one or more of these histone marks may disrupt some aspect of the histone ‘code,’ (Jenuwein and Allis, 2001; Allis and Jenuwein, 2016), which in turn blocks transformation of chromatin into resolved chromosomes. For example, one cause may be an abnormally higher number of histone H3 proteins marked simultaneously with H3K9me2/3 and H3S10p. Normally it is thought that these marks toggle during the transition between heterochromatin and chromosome condensation and resolution, respectively (Fischle et al., 2005). Abnormal retention of H3K9me2/3 may hinder the ‘reading’ of H3S10p by condensins, thereby blocking normal chromosome resolution. In order to fully understand genome elimination by PSR it will be important for future studies to determine how the PCM’s unique chromatin pattern is established and maintained, and how this pattern is translated into some phenotypic outcome.

## How Does PSR Induce Chromatin Changes?

**FIGURE 2:**
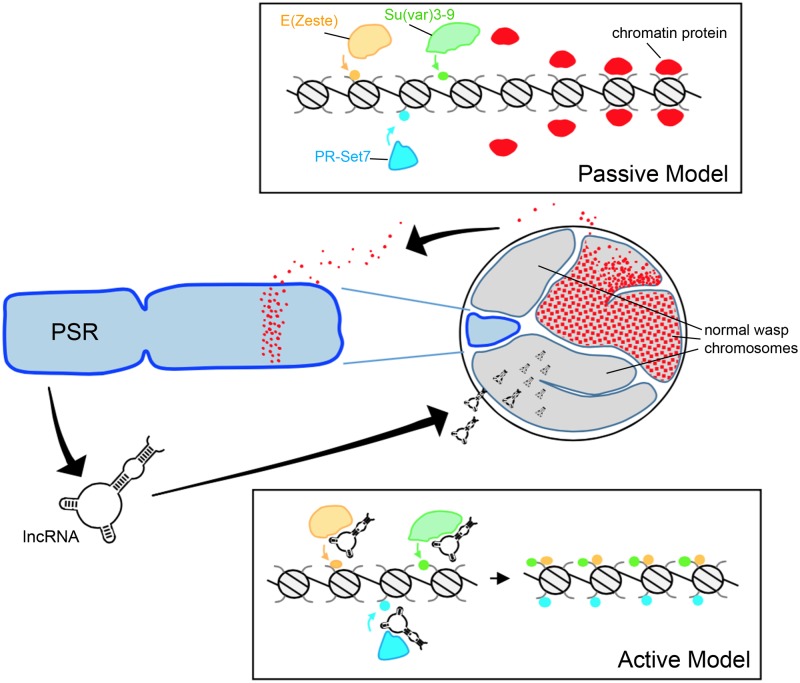
**Hypothetical models for paternal genome elimination by PSR. (Top)** The passive model for genome elimination in which PSR serves as a sink, titrating away one or more chromatin-associated factors that are critical for normal chromosome condensation and resolution. Depletion of these factors allows enzymes such as E(z), Su(var)3-9, and Pr-Set7 to abnormally modify regions of chromatin within the paternal pronucleus with marks such as H3K27me1, H3K9me2,3, and H4K20me1, respectively (also see **Figure 1A**). In the active model **(Bottom)**, one or more PSR expressed factors actively interferes with the chromatin remodeling process. In this example, a PSR-expressed long non-coding RNA (lncRNA) associates with the euchromatin of the wasp’s normal chromosomes (but not PSR). There, these factors inappropriately recruit chromatin-modifying enzymes to these regions, marking the normal chromosomes for elimination. Either of these models could potentially take place during spermatogenesis or in the egg cytoplasm, immediately before the first embryonic mitosis.

As described above, paternal genome elimination involves alterations to the underlying chromatin structure (Swim et al., 2012; Aldrich et al., 2017). Generally speaking, one can imagine that PSR might exert this effect through either passive or active means (**Figure 2**). The passive model, simply put, is that PSR’s presence in the paternal nucleus is enough to interfere with some fundamental chromatin remodeling process of early embryogenesis and that this interference results in genome elimination. Beukeboom and Werren (1993a) proposed a model in which PSR acts as a “sink,” containing an excessive number of binding sites for one or more chromosome resolution factors. When PSR is present, these sites sequester a critical amount of these factors away from the other chromosomes, preventing them from properly resolving (Beukeboom and Werren, 1993a). This type of passive mechanism bears some similarity to meiotic drive found in other B chromosome systems, wherein an expansion of centromeric sequences may allow those chromosomes to outcompete the normal chromosomes for spindle attachments (Fishman and Saunders, 2008).

Any “active” model would require that PSR produce one or more gene products in order to target paternal chromosomes and induce genome elimination. PSR, like many B-chromosomes, is largely composed of repetitive satellite sequences (Eickbush et al., 1992) and until recently little was known about what, if any, genes might be contained therein. Akbari et al. (2013) profiled the testis transcriptome of PSR-carrying and non-carrying males and identified a number of polyadenylated RNAs unique to PSR-carrying males. These sequences were not found within the *N. vitripennis* draft genome (Werren et al., 2010). Moreover, they visually mapped exclusively to PSR, strongly arguing that they are indeed PSR-specific transcripts. The function of these RNAs remains unclear, as does the whether or not they are involved with some aspect of genome elimination. They range in length from ∼500 to ∼1,500 nucleotides, and have low coding potential—the longest potentially coded polypeptide is only 245 amino acids. Furthermore, the RNAs share very little sequence similarity with known protein coding genes—in Nasonia or otherwise—so their functions, if any, are not readily apparent at this time.

As an alternative to encoding proteins, it has been suggested that these PSR-specific transcripts may instead be long non-coding RNAs (lncRNA) (Akbari et al., 2013). Examples of this class of RNA have been identified in diverse species and there is a growing appreciation of the role these RNAs play in a variety of cellular processes (Rinn and Chang, 2012; Kung et al., 2013). One role that is of special relevance to our discussion of genome elimination is the regulation of chromatin by Xist, a mammalian lncRNA involved in dosage compensation via X chromosome inactivation (Brown et al., 1992). Xist accumulates on one of the two X chromosomes and mediates the transcriptional silencing and condensation of that chromosome into a structure called the Barr body. Similar to the PCM, the inactive X is enriched with hypermethylated H3K27 and H4K20 (Plath et al., 2002). Although genome elimination by PSR is evolutionarily unrelated to mammalian dosage compensation, Xist does set a precedent for a possible lncRNA-like role for PSR-produced transcripts in targeting chromatin modifications to the wasp’s normal chromosomes.

## Genome Elimination as a Transgenerational Phenomenon

Genome elimination occurs during the earliest stages of embryogenesis—well before the maternal to zygotic transition (MZT) and the onset of zygotic transcription (Werren et al., 1987; Langley et al., 2014). Given that the PSR chromosome is only carried paternally, any PSR-specific gene involved in genome elimination would have to be expressed prior to fertilization, which presents a problem: how can a gene expressed in one generation induce a phenotype in the next?

Any PSR-expressed gene products involved in genome elimination would need to either exert their effect on the paternal genome during spermatogenesis or somehow be transferred via sperm into the egg. Spermatozoa contain very little cytoplasm and are typically thought of primarily as carriers of DNA (along with a centriole, usually) (Hennig, 1996). Nevertheless, RNA *has* been detected in sperm and recent work has identified a role for such RNAs in mediating transgenerational “paternal effect” phenotypes (Zhao et al., 2006; Rodgers et al., 2013). These discoveries suggest a possible route for PSR-transcribed gene products to be transferred via sperm into the egg where they would be able to participate in paternal genome elimination.

Although histones and other chromatin components can remain associated with DNA through mitosis and meiosis, they are largely removed from the sperm nucleus during spermatogenesis as the paternal DNA is packaged with protamines (Rathke et al., 2007). Furthermore, the PCM’s abnormal modification pattern only appears sometime after fertilization, suggesting that the sperm’s initial chromatin state is largely unperturbed (Aldrich et al., 2017). Although there is evidence in some organisms that a small fraction of histones remains associated with sperm chromatin (Brunner et al., 2014), it seems unlikely that the inheritance of a few modified histones would result in the global effect observed during genome elimination. Nevertheless, as a recent study has shown, discrete epigenetic modification of sperm chromatin can have functional consequences (Siklenka et al., 2015), so this possibility remains a formal mechanism through which paternal chromosomes might be transgenerationally targeted for elimination.

## How Does PSR Avoid Elimination?

In order to be faithfully transmitted to the next generation, PSR must convert female embryos into males via genome elimination while avoiding self-elimination. All paternal chromosomes are indiscriminately targeted for elimination with near-100% efficiency, yet PSR itself somehow escapes this event (Beukeboom and Werren, 1993b; Swim et al., 2012). In order to address this contradiction it is important to more closely examine the features that distinguish PSR from the standard Nasonia chromosomes.

Analyses of cloned sequences from PSR suggest that it is composed largely of repetitive sequences that lack homology to the rest of the *N. vitripennis* genome (Eickbush et al., 1992; Werren et al., 2010). While this observation certainly raises a number of intriguing questions concerning the origin of PSR, it also suggests a potential mechanism for targeting the other chromosomes while avoiding self-elimination. If PSR is targeting specific sequences within the Nasonia genome, then its overall lack of homology might allow it to avoid being targeted and eliminated. Related to this possibility is the observation that PSR either does not have euchromatic arms, or it has a drastically reduced amount of euchromatin relative to normal chromosomes (Reed, 1993). If certain genome elimination factors ‘tract’ to euchromatin specifically, then this characteristic would serve as a means of distinguishing PSR from the other chromosomes.

Recent work has revealed that PSR also differs from paternal chromosomes at the chromatin level. While during metaphase both PSR and the PCM are marked with H3K9me2/3, PSR conspicuously lacks H3K27me1 and H3K20me1 (Aldrich et al., 2017). The mechanism that establishes these chromatin marks remains unknown, as does the means by which such a mechanism would distinguish between PSR and the paternal chromosomes. One possibility may be that PSR’s unique sequence composition is somehow recalcitrant to these particular marks. Regardless, the fact that they are absent from PSR does support a central role for these marks in genome elimination.

## Similarities to Other Chromosome Silencing or Elimination Events

PSR-induced genome elimination is just one of a variety of chromosome silencing and/or elimination events in the eukaryotes. Other similar phenomena include the formation of Barr bodies in mammalian cells (Plath et al., 2002; Rougeulle et al., 2004), the micronucleus of the ciliated protist, *Tetrahymena thermophila* (Duharcourt et al., 2009), and meiotic and mitotic elimination of paternal chromosomes in the fungal gnat, *Sciara coprophila* (Goday and Ruiz, 2002; Greciano and Goday, 2006; Escribá et al., 2011). Although these events resemble PSR in that they involve chromatin-level changes, the specific chromatin modifications in each vary significantly (**Figure 1B**). Furthermore, unlike PSR, all of these events occur as a programmed part of normal development. In contrast, cytoplasmic incompatibility (CI), a conditional male sterility occurring in many arthropods, is induced by an intracellular bacterium called *Wolbachia* (Callaini and Riparbelli, 1996). Like PSR, *Wolbachia* induces genome elimination in order to facilitate its own transmission but to the detriment of the organism (Werren et al., 2008).

While *Wolbachia* is found in both the testis and ovaries, it can only be transmitted maternally within the cytoplasm of the egg. When an infected male mates with an uninfected female, paternal chromatin fails to resolve into distinct chromosomes and is eliminated during early embryogenesis (Callaini and Riparbelli, 1996; Tram et al., 2006). In diploid arthropods such as Drosophila, genome elimination caused by CI results in embryonic lethality, while in Nasonia and other haplodiploids, affected embryos survive but are converted into haploid males (Reed and Werren, 1995; Perrot-Minnot et al., 1996).

Like PSR-induced genome elimination, CI occurs during the first round of embryonic cell division and seems to involve a disruption of the normal chromatin state. Core histones H3 and H4 are improperly loaded onto paternal chromatin and the timing of the first S-phase is prolonged such that replication remains incomplete well after the maternal chromosomes enter into metaphase (Landmann et al., 2009). It is speculated that prolonged and/or incomplete replication prevents chromosome resolution. It is unknown if any specific histone modifications are perturbed by *Wolbachia*, so no direct comparisons can be made to PSR in this regard; however, H4 loading occurs normally in PSR-containing embryos, as does the first round of replication (Swim et al., 2012; Aldrich et al., 2017). This suggests that while the two phenomena share a number of similarities, key mechanistic differences may exist.

## Conclusion

Chromatin plays a central role in regulating a number of fundamental nuclear processes in eukaryotes—including chromosome structure, genome organization, and gene transcription—and is thought to underlie many cases of epigenetic inheritance (Kornberg, 1974; Allis and Jenuwein, 2016). Recent studies in the jewel wasp *N. vitripennis* have identified several distinct chromatin changes associated with targeted paternal genome elimination by the parasitic B-chromosome, PSR (Swim et al., 2012; Aldrich et al., 2017). Although many questions remain concerning the mechanism through which the paternal genome is targeted and eliminated, it is likely that PSR is either coopting or disrupting a fundamental chromatin-related process that is unique to the paternal genome during the earliest phases of embryogenesis. Therefore, future studies in this area may shed light on other chromatin-based regulatory phenomena either by elucidating common pathways or by highlighting the diverse mechanisms that can result in chromosome/genome elimination or silencing.

## Author Contributions

All authors listed, have made substantial, direct and intellectual contribution to the work, and approved it for publication.

## Conflict of Interest Statement

The authors declare that the research was conducted in the absence of any commercial or financial relationships that could be construed as a potential conflict of interest.
